# Atherogenic circulating lipoproteins in ischemic stroke

**DOI:** 10.3389/fcvm.2024.1470364

**Published:** 2024-12-06

**Authors:** Sonia Benitez, Núria Puig, Pol Camps-Renom, José Luis Sánchez-Quesada

**Affiliations:** ^1^ Cardiovascular Biochemistry Group, Institut de Recerca Hospital de Sant Pau (IR Sant Pau), Barcelona, Spain; ^2^ CIBER-Diabetes and Metabolic Diseases (CIBERDEM), Madrid, Spain; ^3^ Stroke Unit, Department of Neurology, Hospital de La Santa Creu I Sant Pau, IR Sant Pau, Barcelona, Spain

**Keywords:** ischemic stroke, oxidized LDL, small dense LDL, electronegative LDL, HDL, carotid atherosclerosis

## Abstract

The fundamental role of qualitative alterations of lipoproteins in the early development of atherosclerosis has been widely demonstrated. Modified low-density lipoproteins (LDL), such as oxidized LDL (oxLDL), small dense LDL (sdLDL), and electronegative LDL [LDL(-)], are capable of triggering the atherogenic process, favoring the subendothelial accumulation of cholesterol and promoting inflammatory, proliferative, and apoptotic processes characteristic of atherosclerotic lesions. In contrast, high-density lipoprotein (HDL) prevents and/or reverses these atherogenic effects. However, LDL's atherogenic and HDL's anti-atherogenic actions may result altered in certain pathological conditions. The molecular mechanisms underlying the impaired effects of altered lipoproteins have been studied in numerous *in vitro* and *in vivo* studies, and have been extensively analyzed in coronary atherosclerosis, especially in the context of pathologies such as dyslipidemia, diabetes, obesity, and metabolic syndrome. However, the corresponding studies are scarcer in the field of ischemic stroke, despite carotid arteriosclerosis progression underlies at least 20% of ischemic strokes. The present review relates qualitative alterations of LDL and HDL with the development of carotid arteriosclerosis and the occurrence of ischemic stroke.

## 1 Atherosclerotic cardiovascular disease

Cardiovascular (CV) diseases, responsible for more than 17.5 million deaths per year worldwide, represent a health and socioeconomic problem of huge magnitude. Increased CV disease prevalence in recent decades is largely due to the higher incidence of CV risk-associated pathologies, such as diabetes, dyslipidemia, obesity, and hypertension, with population ageing, unhealthy eating habits, and sedentary lifestyles undoubtedly acting as contributing factors (1).

Early development of atherosclerosis is a main underlying cause of CV disease, resulting in a condition known as atherosclerotic CV disease (ASCVD). Atherosclerosis is defined as a thickening and loss of elasticity in the arterial wall of large- and medium-sized arteries, and particularly in zones of curvature or bifurcation with turbulent flows, leading to a narrowing (stenosis) of the vascular lumen (2). Low-shear stress zones are susceptible to atherosclerotic plaque development, with rupture eventually leading to reduced blood flow and the onset of CV events (3). Vessels more prone to developing atherosclerosis are the abdominal aorta, and peripheral, coronary, and carotid arteries, leading, respectively, to the following forms of ASCVD: aortic atherosclerotic disease, peripheral artery disease (claudication), coronary heart disease (myocardial infarction, angina pectoris), and cerebrovascular disease (transient ischemic attack, ischemic stroke).

Atherosclerosis is a long-term process that is initially triggered by lipoprotein entry and retention in the artery wall, leading to intracellular and extracellular lipid accumulation in the subendothelial space. The progressive and slow deposition of lipids, occurring in parallel with an inflammatory response and monocyte recruitment from circulating blood to the arterial intima, together result in a narrowing of the artery wall, referred to as atherosclerotic plaque (2).

**Figure 1:**
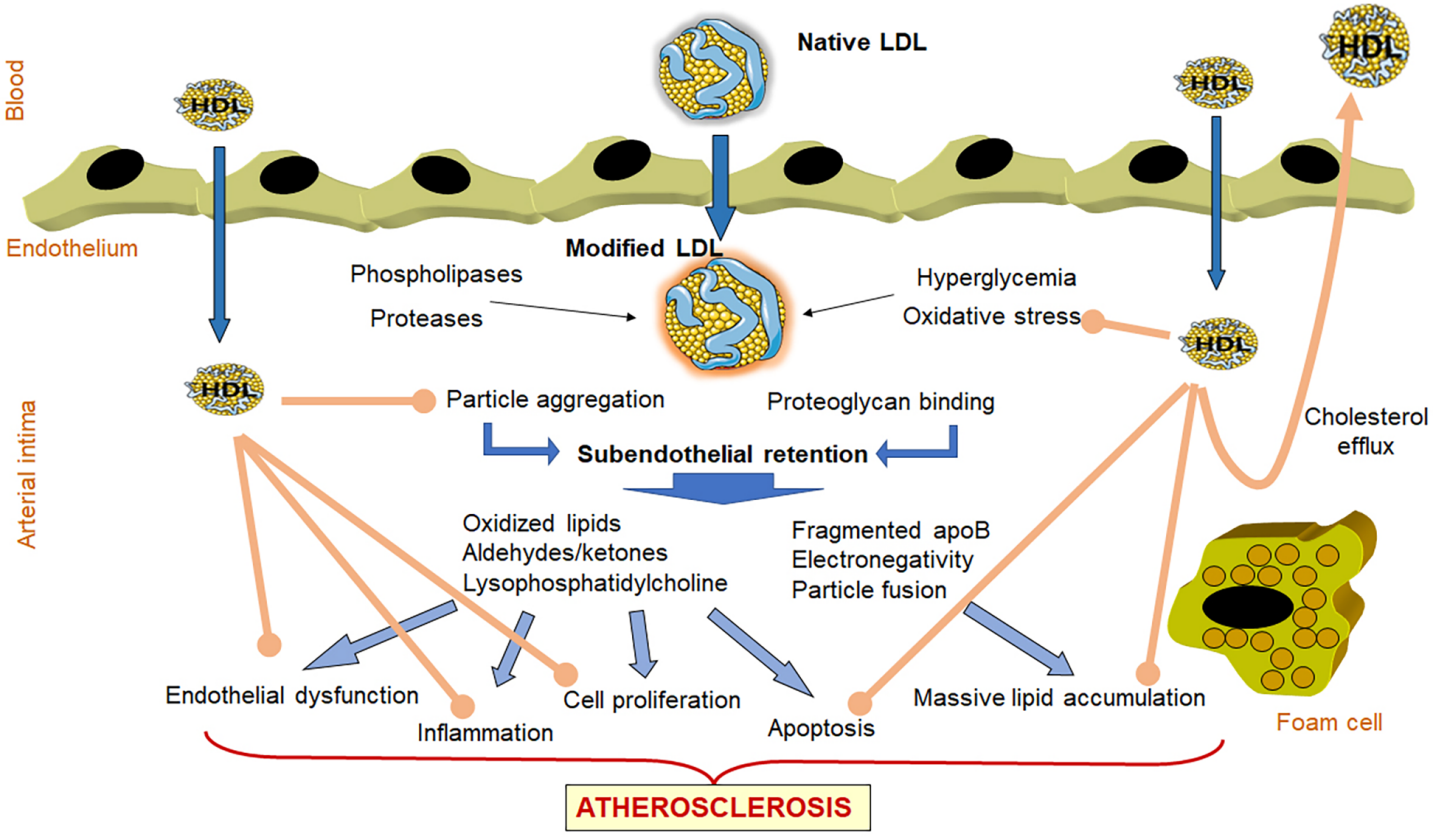
Role of LDL and HDL in atherogenesis. LDL entry to the microenvironment of the subendothelial space favors chemical modifications resulting from oxidative stress and the actions of lipolytic and proteolytic enzymes. These modifications favor LDL aggregation and increased binding to proteoglycans, facilitating its subendothelial retention. Retained modified LDL particles induce endothelial dysfunction, the recruitment of leukocytes with enhanced inflammatory response, the differentiation of monocytes into macrophages, the emergence of apoptotic processes, and the formation of lipid-loaded foam cells. Together, all of these events lead to the development of the atherosclerotic plaque. The atherogenic effects of LDL are indicated with blue arrows. In contrast, HDL particles that enter in the arterial wall can prevent LDL oxidation and aggregation, and display anti-inflammatory, antiproliferative and antiapoptotic properties (brown arrows). In addition, HDL induces the efflux of cholesterol from lipid-loaded foam cells and returning the excess of cholesterol to blood circulation for its metabolization in the liver.

Low-density lipoprotein (LDL) plays a major role in different stages of the atherosclerotic plaque formation (Figure 1) (4). LDL entry to the microenvironment of the subendothelial space favors chemical modifications resulting from oxidative stress and the actions of lipolytic and proteolytic enzymes. Modified LDL, with the acquired pro-atherogenic properties, favors subendothelial retention and induces endothelial dysfunction, the recruitment of leukocytes with enhanced inflammatory response, the differentiation of monocytes into macrophages, the emergence of apoptotic processes, and the formation of lipid-loaded foam cells (5–7). To attract leukocytes to the lesioned area, modified LDL promotes the expression of adhesion molecules and chemokines by endothelial cells. In monocyte-derived macrophages, modified LDL promotes the release of more chemokines and cytokines, such as tumor necrosis factor alpha (TNF-α) and interleukin-1 (IL-1β), in addition to growth factors. This response contributes to the proliferation and activation of smooth muscle cells (SMCs), which together with macrophages, uptake modified LDL becoming lipid-loaded foam cells, a hallmark of atherosclerosis (8, 9).

Contrasting with LDL, high-density lipoprotein (HDL) is considered athero-protective (Figure 1), owing to its capacity to induce cholesterol efflux from tissues (10, 11) and to its anti-oxidant and anti-inflammatory properties (12). Of note, HDL's effects include the inhibition of LDL modification and mitigation of the atherogenic effects of modified LDL (12, 13).

The contribution of LDL cholesterol (LDL-C) to CV risk has been extensively studied, given its role in progression of atherosclerosis (14), which contrasts with the inverse association with vascular risk of HDL cholesterol (HDL-C) (15). However, the fact that therapies to regulate HDL-C or LDL-C levels do not eliminate vascular risk would suggest that, over and above concentration levels, the actual quality of lipoproteins is crucial to determining susceptibility to CV disease development.

## 2 Ischemic stroke and blood lipoprotein levels

Stroke represents the third cause of mortality in western countries and is a leading cause of disability and dementia worldwide. Furthermore, its impact is aggravated by the fact that around 20% of patients experience recurrence within 5 years of follow-up, increasing the risk of severe disability (16). Around 80% of strokes are ischemic in origin (17), and in approximately 20% of those cases, the cause is large-vessel atherosclerosis (18), referred to as atherothrombotic stroke (19). Plaques in the internal carotid artery are most frequently involved in this subtype (20), with stroke triggered by plaque rupture leading to thrombus formation and subsequent distal embolism (3), and/or by hemodynamic insufficiency, possibly attributable to progressive vessel occlusion caused by atherosclerotic plaque development (21).

The distribution of the modifiable vascular risk factors differs according to the type of stroke and the patient's sex and age (22). Stroke incidence is higher in women than in men for those younger than 30 years, whereas rates are higher in men during midlife. In subjects older than 80 years the incidence is slightly higher in women. Regarding carotid stenosis, women have a higher degree of stenosis, but less carotid plaque area (23) and less vulnerable plaques with lower risk than men (24, 25).

In atherothrombotic stroke, the recurrence risk of major vascular events is much higher than for other stroke subtypes (26), for which reason secondary prevention is essential. The main therapeutic approach is carotid endarterectomy, implemented according to the degree of stenosis (27). In addition to luminal narrowing, the inflammatory state and lipid content of the plaque are considered key factors in determining carotid plaque vulnerability. As explained above, lipoproteins play a key role in the progression of atherosclerosis, with LDL—most particularly when modified in the arterial wall—acting as a stimulus that strongly promotes inflammation and lipid accumulation (5).

Although elevated serum lipids and lipoproteins have been extensively associated with CV disease, there is little information regarding cerebrovascular disease. However, coronary patients are reported to have an increased risk of stroke (28), and the relationship between lipids/lipoproteins and ischemic stroke, and particularly atherothrombotic stroke, has been addressed in several epidemiological studies.

High plasma levels of total cholesterol, and particularly of LDL-C, have been associated with atherothrombotic stroke (29, 30) and progressive carotid stenosis (31). In line with those findings, lipid-lowering therapy to decrease total cholesterol and LDL-C levels has been shown to mitigate the progression of carotid atherosclerosis (32, 33). According to the SPARCL study (Stroke Prevention by Aggressive Reduction in Cholesterol Levels), high-dose statins reduce the overall occurrence of strokes and CV events, with only a slight increase in the rate of hemorrhagic strokes (34). Likewise, PCSK9 inhibitors have been reported to reduce the incidence of stroke by 25% (35). Although some studies have pointed to an association between aggressive LDL-C-lowering therapies and an increased risk of hemorrhagic stroke, this association has not been conclusively demonstrated (36).

Notably, Sniderman et al. (37) argued that actual quantification of LDL particles or of apolipoprotein (apo) B was more important than LDL-C plasma levels in coronary artery disease. For ischemic stroke, the risk attributable to elevated apoB or non-HDL-C has been reported to be higher than the risk attributable to elevated LDL-C (38).

Elevated levels of lipoprotein(a) [Lp(a)] are postulated to contribute to so-called residual CV risk, i.e., risk not directly derived from elevated LDL-C (39, 40). Lp(a) consists of an LDL molecule that also contains the apolipoprotein (a) [apo(a)], covalently linked to apoB-100. Apo(a) is formed by multiple repetitions of structures called kringles, and depending on the number of repetitions differ in the CV risk, having small apo(a) isoforms a greater CV risk compared to larger isoforms (41). Higher Lp(a) concentrations are independently associated with long-term ASCVD risk and may amplify CV risk when concomitant with carotid plaque (42). Referring specifically to ischemic stroke, Lp(a) may play an especially relevant role, since its antifibrinolytic action may be determinant in the surge of thrombotic processes leading to an ischemic event (43, 44). Besides this antifibrinolytic action, the atherogenic effect of Lp(a) seems to be related to its ability to bind highly inflammatory oxidized phospholipids. These phospholipids include a soluble fraction in the surface of the lipoprotein particle and a fraction of these phospholipids covalently bound to apo(a) (45, 46). Indeed, Lp(a) is the main plasma transporter of oxidized phospholipids (47). Consequently, Lp(a) could play a role in the oxidative stress associated with the development of arteriosclerosis (48) and, therefore, be related to the oxidative modifications of LDL described in the following sections, which play a prominent role in the early appearance of ischemic events. Elevated Lp(a) levels have been associated with a high incidence of ischemic stroke in most (49–51)—but not all (52)—studies, and have also been associated with a greater vascular event recurrence risk in patients with acute first-ever ischemic stroke (53) and with vulnerable carotid plaque and plaque development (54, 55). The association of Lp(a) with carotid plaque vulnerability is sex-specific, since it was associated with presence of intraplaque hemorrhage in women and with stenosis degree in men (54). A recent meta-analysis concluded that increased Lp(a) concentrations could be considered a predictive marker for identifying individuals at risk of developing ischemic stroke (56).

Triglycerides may be independently associated with the risk of ischemic stroke (57), while elevated remnant cholesterol, i.e., the cholesterol transported in triglyceride-rich lipoproteins, has likewise been associated with a higher risk of ischemic stroke in the general population (58). A recent genome-wide association study (GWAS), using Mendelian randomization, has suggested that remnant cholesterol is causally associated with large-artery atherothrombotic stroke (59). Moreover, remnant cholesterol levels in patients with ischemic stroke have been positively associated with carotid artery intima-media thickness (60), with the concentration of triglyceride-rich particles predicting the presence of vulnerable carotid plaque independently of LDL-C (61, 62).

Despite the well-known inverse association between HDL-C levels and coronary heart disease, findings have not been so consistent for stroke. HDL-C levels are weakly or not at all associated with the incidence or prevalence of ischemic events, and do not predict recovery from stroke. However, some studies have shown an inverse association in the specific case of atherothrombotic stroke (63, 64). As occurs with LDL, the HDL particle count is a promising risk prediction parameter. While most studies to date have been single-cohort studies assessing only coronary disease or composite vascular outcomes, a study by Singh et al. (65) has reported that HDL particles is a robust marker for ischemic stroke in the overall population, and also that it inversely correlates with both myocardial infarction and stroke, albeit displaying racial disparities.

Conflicting results regarding the predictive power of lipid and lipoprotein blood levels for stroke may be partly due to the time of blood collection in patients, because, as already described in the 1980s (28), stroke itself exerts a lipid-lowering effect. Growing evidence suggests that—in addition to lipoprotein quantitative parameters—certain lipoprotein component concentrations and functional alterations are relevant to the pathology of ischemic stroke, and particularly to the atherothrombotic subtype.

## 3 Ischemic stroke and qualitative lipoprotein changes

Despite the association between plasma lipoprotein levels and ASCVD (including atherothrombotic stroke), mounting evidence points to the fact that lipoprotein concentration is not always the key, as the presence of atherosclerosis is not exclusively determined by quantitative lipid parameters such as high LDL-C levels (66). The importance of other lipoprotein-related factors is suggested by subjects with normal LDL-C still having residual CV risk and by a diminished association with LDL-C when adjusting for other lipoproteins (67, 68). Those observations are likely based on the fact that circulating lipoproteins comprise subclasses that are heterogeneous in size, density, composition, and function, and with different involvement in ASCVD.

Alterations in HDL and LDL particle size in ischemic stroke occur parallel to changes in chemical composition, as reported in several studies (69–74). Just beginning to be deciphered is the specific lipid and protein composition of lipoproteins involved in atherothrombotic stroke, with interesting preliminary data obtained from lipidomic and proteomic studies of lipoproteins isolated from ischemic patients. Lepedda et al. (72) showed increased acute-phase serum amyloid A (SAA) levels in all lipoprotein fractions obtained from the patients in their study, while a proteomic study by Finamore et al. (71) revealed that mainly LDL, but also HDL, showed a higher content of proteins associated with inflammation, immunity, and coagulation, and specific protein signatures for patients with hypoechoic plaques. Regarding lipids, a preliminary lipidomic study in lipoproteins isolated from patients who had undergone carotid endarterectomy showed lipid alterations, particularly in specific phospholipids in the LDL of patients with hypoechoic plaques (73). It is very feasible to consider that such physical and chemical alterations in LDL might lead to a higher prevalence of modified LDL particles in ischemic stroke. The role of modified LDL and the influence of altered qualitative properties of HDL, specifically in relation to atherothrombotic stroke, are discussed in the following sections.

### 3.1 Qualitative LDL changes: modified LDL

**Figure 2:**
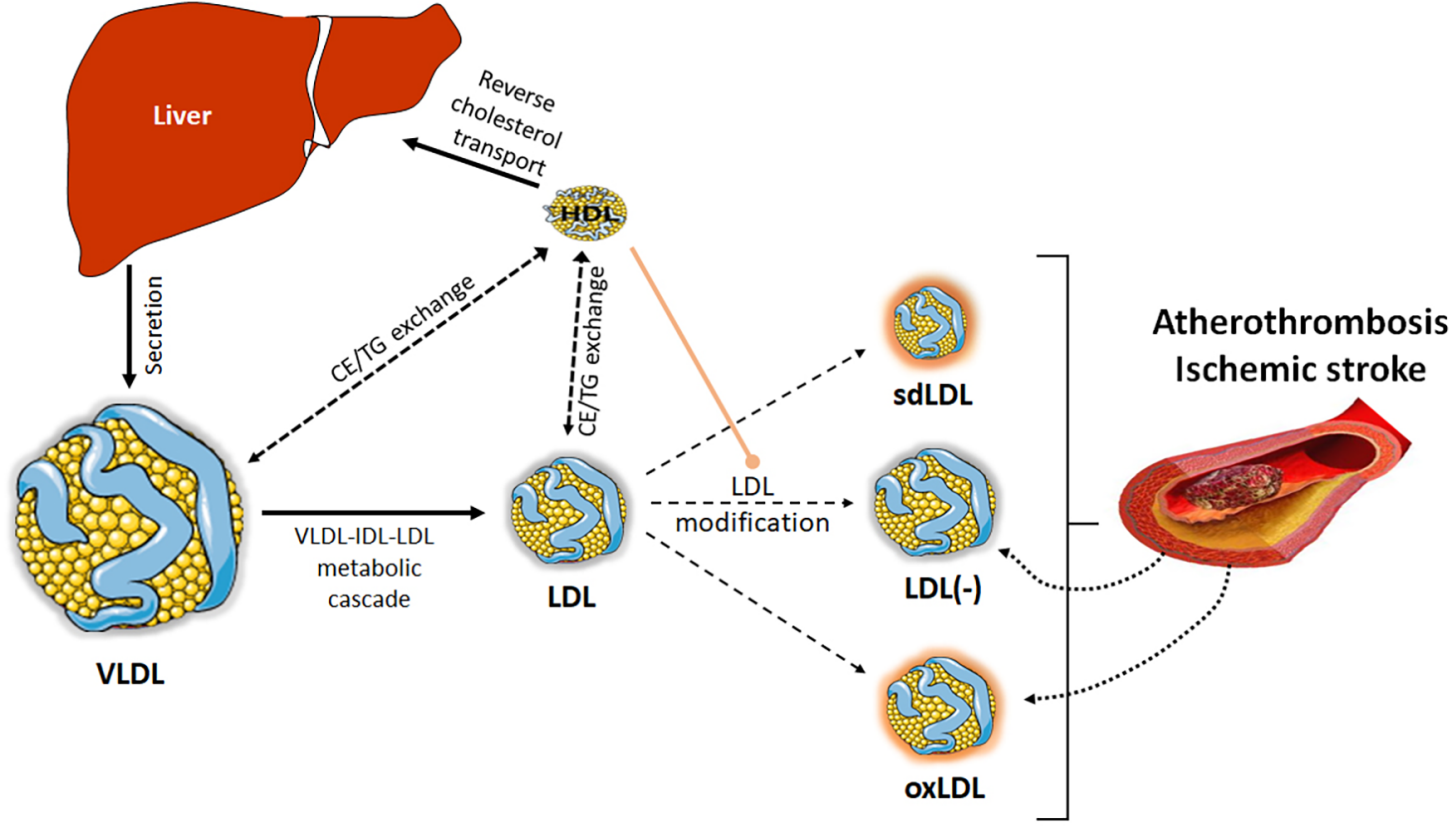
LDL modification. Besides the modification of LDL in the arterial wall, LDL particles can also suffer different alterations in blood, leading to modified LDL particles. Alterations in VLDL-IDL-LDL catabolic cascade or in the lipid transfer processes among lipoproteins can favor the formation of sdLDL or LDL(-). Other processes, such as overload of non-esterified fatty acids or oxidative phenomena occurring in blood can also lead to the formation of LDL(-) or oxLDL. All these modified LDL particles will contribute to the development of atherosclerosis. In turn, the rupture of the atherosclerotic plaque after an ischemic event can release its content of modified lipoproteins, thereby contributing to the plasma pool of LDL(-) and oxLDL. For its part, HDL plays an antiatherogenic role, preventing the formation of modified LDL, through its antioxidant capacity or the ability to capture non-esterified fatty acids.

The presence of modified LDL is pivotal to determine susceptibility to atherosclerosis and vulnerable lesions. Alterations in LDL catabolism or other chemical processes favor the LDL modification and abnormal LDL particle formation in terms of composition, size, and electric charge (Figure 2). Specifically referring to atherothrombotic stroke patients, Yatsu et al. (75) reported that their monocyte-derived macrophages displayed a reduced ability to scavenge modified LDL. This fact, together with increased levels of modified LDL forms and the impaired protective effects of HDL, may contribute to rapid disease progression. In this context, while the role of oxidized LDL (oxLDL) in ischemic stroke has been extensively evaluated (76), less studied is the importance of a modified form of LDL with a negative charge, called electronegative LDL [LDL(-), also known as L5], found in circulation (77). The roles played by modified LDL in the form of oxLDL and LDL(-), and also by small dense LDL (sdLDL), are discussed below.

#### 3.1.1 Oxidized LDL

It is widely accepted that LDL can be modified by oxidation in the subendothelial space, where it is exposed to free radicals and oxidative enzymes released by environment cells. This has led to thinking that oxLDL detected in plasma comes from the subendothelial space after plaque rupture. Accordingly, most studies analyzing oxLDL association with ischemic stroke have reported that oxLDL levels in blood are higher in patients in the acute ischemic phase than in healthy controls (78–81), and are reduced by post-event statin therapy (80, 82). Moreover, for patients with acute ischemic stroke, Wang et al. (83) found a relationship between oxLDL plasma levels and the National Institute of Health Stroke Scale score. Blood concentrations of oxLDL are reported to be particularly increased in patients with large-artery atherosclerosis compared with other stroke subtypes (79, 80, 84), and have also been associated with lacunar stroke in small vessels (85, 86). The concentration of oxLDL in plasma and in the atherosclerotic plaque has been associated with the vulnerability of carotid plaque (87), although no association was found in a recent study (78).

Those studies carried out in the acute ischemic phase do not clarify whether the elevated oxLDL levels are a consequence or cause of the stroke. However, several prospective studies have suggested that, as well as being associated with the acute stroke phase, oxLDL plays a role in the future occurrence of vascular events (88–91) and in 1-year recurrence (92), and is also associated with poorer patient cognitive and functional evolution (93, 94). In symptomatic patients, some studies have related oxLDL with transient ischemic attack (89, 95). High levels of oxLDL and high-sensitivity C-reactive protein (CRP), when combined, are associated with increased risk of recurrent stroke, combined vascular events, and poor functional outcome in patients with minor stroke or transient ischemic attack (94).

Taken together, those studies not only demonstrate a seemingly solid relationship between elevated oxLDL levels and ischemic stroke onset and recurrence, but also point to plasma oxLDL as a putative predictor of stroke in asymptomatic cases.

#### 3.1.2 Electronegative LDL

LDL(-), a heterogeneous entity that encompasses several LDL forms modified by different mechanisms and having an increased negative charge in common, is a minor plasma form of modified LDL with atherogenic properties (96–100). Among other atherogenic properties, LDL(-) has increased susceptibility to aggregate and to bind to proteoglycans, inducing inflammation, apoptosis, and cell proliferation in several arterial wall cell types (101–103). LDL(-), which constitutes about 3%–5% of total LDL in healthy subjects, is present in higher proportions in pathologies associated with vascular risk, including dyslipidemia and diabetes (104), and in subclinical atherosclerosis in association with the degree of carotid stenosis (105, 106). In metabolic syndrome, LDL(-) levels were higher in men than in premenopausal women, which can contribute to their higher predisposition to CV risk (107). LDL(-) is also increased during the acute phase of vascular events such as myocardial infarction (108) and ischemic stroke (78, 109).

Of 2 studies focused on LDL(-) in ischemic stroke, only one was specifically conducted in patients with carotid atherosclerosis (78). Despite a normal lipid profile, that population with recent ischemic stroke showed higher blood levels of LDL(-) and oxLDL than control subjects. The favorable lipid profile may be attributable to the known drop in lipid levels and to the administration of high-dose statins after the ischemic event. Importantly, the proportion of LDL(-), but not of oxLDL, has been associated with carotid plaque features ascribed to vulnerability, particularly the degree of carotid stenosis, hypoechogenicity, and diffuse intraplaque neovascularization (78). This would suggest that LDL(-) may be a marker of plaque vulnerability in ischemic stroke associated with carotid atherosclerosis, as proposed by Shen et al. (109); in the latter study, conducted in patients with acute ischemic stroke, higher values of LDL(-) than in the study of Puig et al. (78) were reported, presumably owing to differences in the population, time of blood extraction, and the chromatographic method of LDL(-) isolation. Interestingly, that study suggested that LDL(-) triggers ischemic stroke by promoting thrombosis, through the induction of platelet aggregation and hemostasis via lectin-like oxidized LDL receptor-1 (LOX-1) and IκB kinase 2 (IKK2)/nuclear factor–κB (NF-κB) signaling (109). The authors suggest that LDL(-)-induced platelet activation promotes their aggregation and αβ amyloid peptide release, leading to increased platelet reactivity and stroke complications.

Interestingly, the roles of LDL(-) and Lp(a) have recently been compared in regard to ASCVD development in a study (110), which indicates that both atherogenic lipoproteins contribute to residual CV risk through different mechanisms.

#### 3.1.3 Small dense LDL

As compared with large LDL particles, prevalence of sdLDL particles is strongly associated with early development of CV disease. While sdLDL, as generated by metabolic alterations in subjects with hypertriglyceridemia (111), cannot be considered as a modified form of LDL, it is closely related to modified LDLs such as oxLDL, glycosylated LDL, and LDL(-) for 2 reasons. First, sdLDL is prone to modification by oxidation or glycosylation, favoring the formation of oxLDL and glycosylated LDL (112, 113). And second, sdLDL has a slightly increased electronegative charge compared with large and intermediate LDL particles, so a part of LDL(-) is made up of sdLDL particles. In fact, LDL(-) is smaller and more dense in normolipidemic and hypertriglyceridemic subjects (114, 115).

In the field of stroke, several studies have associated the prevalence of sdLDL particles with an increased risk of ischemic stroke (74, 116, 117) and with neuroimaging markers of cerebral small vessel disease (118), an association shown to be particularly robust in a large prospective study conducted in the general population (119). Moreover, a recent study has reported that higher sdLDL cholesterol (sdLDL-C) levels are associated with an increased risk of incident carotid plaques and especially vulnerable plaques, even in patients with normal LDL-C values (120). sdLDL has also been associated with poor prognosis after stroke (121).

### 3.2 Qualitative HDL changes

While the inverse association between HDL-C and coronary heart disease is widely accepted, the causal relation between HDL and atherosclerosis has not yet been fully elucidated. Therapeutic approaches to raising HDL-C have not been as effective as expected in lowering CV risk (122). The hypothesis that HDL-C concentration is the only factor determining the beneficial role of HDL is now considered questionable, and is gradually being replaced by the hypothesis that HDL functionality encompasses several physiological functions beyond cholesterol efflux that are essential to determining protection against atherosclerosis (123).

For pathological and inflammatory conditions, it is well established that biochemical changes in HDL are associated with dysfunctional HDL and the development of ASCVD (124, 125). The main alterations in HDL qualitative properties described for patients with ischemic stroke are summarized in Table 1. A number of studies have described the presence of large HDL particles in patients with ischemic stroke (69, 70, 126), with some patients also showing biochemical HDL alterations leading to impaired functionality at different levels: cholesterol efflux ability (126), endothelial cell protection (69), antioxidant capacity (70, 127), and anti-inflammatory potential (70).

**Table 1:** Altered HDL features in patients with ischemic stroke.

Altered feature	Phase		Reference
	Acute <24 h	>24 h	
Protein content			
↑ apoE, apoJ ↓ apoA-IV	x	x	Plubell et al. (126)
↓ apo A-I	x		Ortiz-Muñoz et al. (69)
↑ Inflammatory molecules: SAA2 SAA1	x	x x	Plubell et al. (126)
			Lepedda et al. (72)
↓ Paraoxonase-1 and other alterations in enzymes and proteins	x		Ortiz-Muñoz et al. (69)
	x		Varela et al. (70)
	x	x	Plubell et al. (126)
Size			
Larger size	x		Ortiz-Muñoz et al. (69)
	x	x	Plubell et al. (126)
Anti-atherogenic properties			
↓ Cholesterol efflux	x	x	Plubell et al. (126)
↓ Endothelial cell protection	x		Ortiz-Muñoz et al. (69)
↓ Anti-oxidant ability	x		Varela et al. (70)
	x		Damayanthi et al. (127)
↓Aanti-inflammatory properties	x		Ortiz-Muñoz et al. (69)
	x		Varela et al. (70)

The loss of an anti-atherosclerotic function in HDL in ischemic patients has been associated with alterations in the protein cargo, which includes several apos, but also proteins that participate in acute phase response and platelet activation, among them: apoE, apoA-IV, apoJ, apoF, apoL1, apoM, apoC-IV, α-1-antitrypsin, inter-α-trypsin inhibitor, paraoxonase-1, anthrax toxin receptor-2, serpina1, prenylcysteine oxidase-1, and SAA (72, 126). Decreased apoA-I in HDL from ischemic patients probably accounts for loss of functionality, according to Ortiz-Munoz et al. (69), who suggested that, in parallel with diminished apoA-I content, HDL displays fewer protective actions on endothelial cells. HDL particle size seems to impact on HDL function and on stroke outcome, with large HDL particles associated with both diminished anti-oxidant and anti-inflammatory properties and unfavorable outcomes (70).

Other authors have found associations between HDL subspecies and both specific apo content patterns (apoA-I, apoC-III, ApoE, apoJ) and vascular brain injury, including both covert and overt brain infarcts (128). HDL protein alterations in ischemic stroke patients have been attributed to proteome remodeling, owing to the existing inflammatory milieu in the acute phase (126). This interesting study by Plubell et al. describes that changes in HDL proteins in the early acute phase are associated with stroke recovery.

Besides protein cargo, it is well stablished that lipid composition has impact on the quality of HDL. For instance, the relative composition in triglycerides and cholesterol esters of the lipid core affects the conformation of apoA-I and the ensuing antioxidant activity of HDL (129, 130). Regarding lipids in the surface of HDL, the content of non-esterified cholesterol modulates the fluidity of lipoprotein surface, which has a direct effect on the oxidizability of HDL (129). Also, phospholipids and their fatty acid composition have an impact on anti-inflammatory activity of HDL (131). Sphingomyelin content influences HDL's efflux capacity (132). Ceramides, independently, or as precursors of sphingosine 1 phosphate (S1P), also have a relevant role in HDL function (133). S1P confers to HDL atheroprotective properties, including its capacity against apoptosis (134), inflammation (135) and vasodilatation (136, 137). However, contrariwise to LDL, little is known about lipid alterations in HDL in ischemic stroke, and further research is needed. Only the study by Nieddu et al. (73) has analyzed HDL lipidomics in the context of ischemic stroke, but the main differences were observed in LDL (as discussed before), not in HDL.

As occurs with LDL, an increased negative electric charge of HDL has also been also associated with HDL functionality (138), with a number of studies reporting the presence of HDL with increased negative charge, named H5 or HDL(-), in inflammation-related diseases (139–141). These negative HDL particles have been shown to impair cholesterol efflux and anti-inflammatory and anti-apoptotic actions, and to even promote inflammation and foam cell formation (140). An increased presence of oxidized HDL (oxHDL) has also been reported in both atherosclerotic plaques and blood circulation in several diseases, including acute myocardial infarction (141, 142). Accordingly, any increase in H5 or oxHDL in ischemic stroke, and particularly in atherothrombotic stroke, merits further investigation.

## 4 Lipoprotein-based therapies to prevent ischemic stroke

The main lipid-related therapy to reduce ischemic stroke risk is statin administration, whose main beneficial effects—derived from their inhibition of HMG-CoA reductase—are to reduce cholesterol biosynthesis and modulate lipid metabolism. Statins can also improve the biological characteristics of lipoproteins, by changing their chemical composition and decreasing oxLDL and LDL(-) levels (143–145). The additional fact that statins exert pleiotropic and anti-atherosclerotic effects, independently of their hypolipidemic action, significantly contributes to reduce CV event and mortality rates, with greater benefits in patients at high risk (146). Regarding ischemic stroke, several studies [summarized in (147)] have reported statins to reduce the ischemic stroke risk without increasing the hemorrhagic stroke risk.

Other more recently developed lipid-lowering agents demonstrate strong efficacy and are useful to prevent ASCVD. For patients at very high or high CV risk who are not responsive to or are intolerant of statins, ezetimibe, alone or in combination with statins, and PCSK9 inhibitors may reduce stroke risk (148). In the FOURIER study of patients with established atherosclerosis, PCSK9 inhibition with evolocumab added to statins reduced the risk of ischemic stroke (149). Bempedoic acid is a new lipid-lowering medication for the prevention and treatment of CV disease; currently in Phase III clinical trials (150), its effect on ischemic stroke is still pending of evaluation. Likewise, not yet tested for ischemic stroke is icosapent ethyl, an omega-3 fatty acid with triglyceride-lowering action that has shown promising results in reducing plasma triglyceride levels and major adverse CV events (151).

Given that lipoprotein function more than concentration plays a key role in ischemic stroke, future studies should address the qualitative properties of LDL and HDL and strategies to mitigate the generation of oxLDL and LDL(-). In this regard, the ability of statins to normalize alterations in lipoprotein composition and size and to reduce elevated modified lipoprotein levels is well known (152, 153). A recent study has reported that PCSK9 inhibition lowers LDL aggregation susceptibility, an LDL modification associated with future CV-related death (154). Antibodies against LDL(-) with athero-protective action have been detected in human and murine model blood and atherosclerotic plaques (155, 156), and based on one of those antibodies, a peptide with inflammatory properties has been designed that has potential to generate vaccines to immunize against LDL(-) and prevent atherosclerosis (157).

In sum, further investigations focused on the qualitative properties of lipoproteins and overcoming their impaired functioning are essential to understanding and preventing ischemic stroke, and particularly the atherothrombotic stroke subtype.

## 5 Conclusions

The particularities of ischemic stroke of atherothrombotic origin make it necessary to further explore alterations in lipoprotein functioning in stroke patients. In contrast with coronary disease, where these alterations have been extensively studied, for ischemic stroke, relatively few studies have explored abnormal lipoprotein functioning. The fact that quantitative alterations in the lipid profile are less frequent in patients with ischemic stroke than in patients with coronary atherosclerosis would suggest that in the former qualitative lipoprotein alterations may play a key role. In this context, to decipher the contribution of such alterations to this disease and to determine the molecular mechanisms involved poses a challenge in terms of designing new therapies addressed at preventing ischemic stroke.

## Author's note

SB and JS-Q are members of the CIBER of Diabetes and Metabolic Diseases (CIBERDEM; CB07/08/0016). S.B. is member of EpiLipidNET COST action. SB and PC-R are members of RICORS-ICTUS (RD21/0006/0006). SB, JS-Q and NP are members of the Quality Research Group 2017-SGR–1149 of the Generalitat de Catalunya, and of the Vascular Biology Working Group of the Spanish Society of Atherosclerosis. IR Sant Pau is accredited by CERCA Programme/Generalitat de Catalunya.
