# Supplementary figures and images for: Atherogenic circulating lipoproteins in ischemic stroke
Source: Front Cardiovasc Med. 2024 Dec 6;11:1470364. doi: 10.3389/fcvm.2024.1470364 (PMC11659270; doi:10.3389/fcvm.2024.1470364)

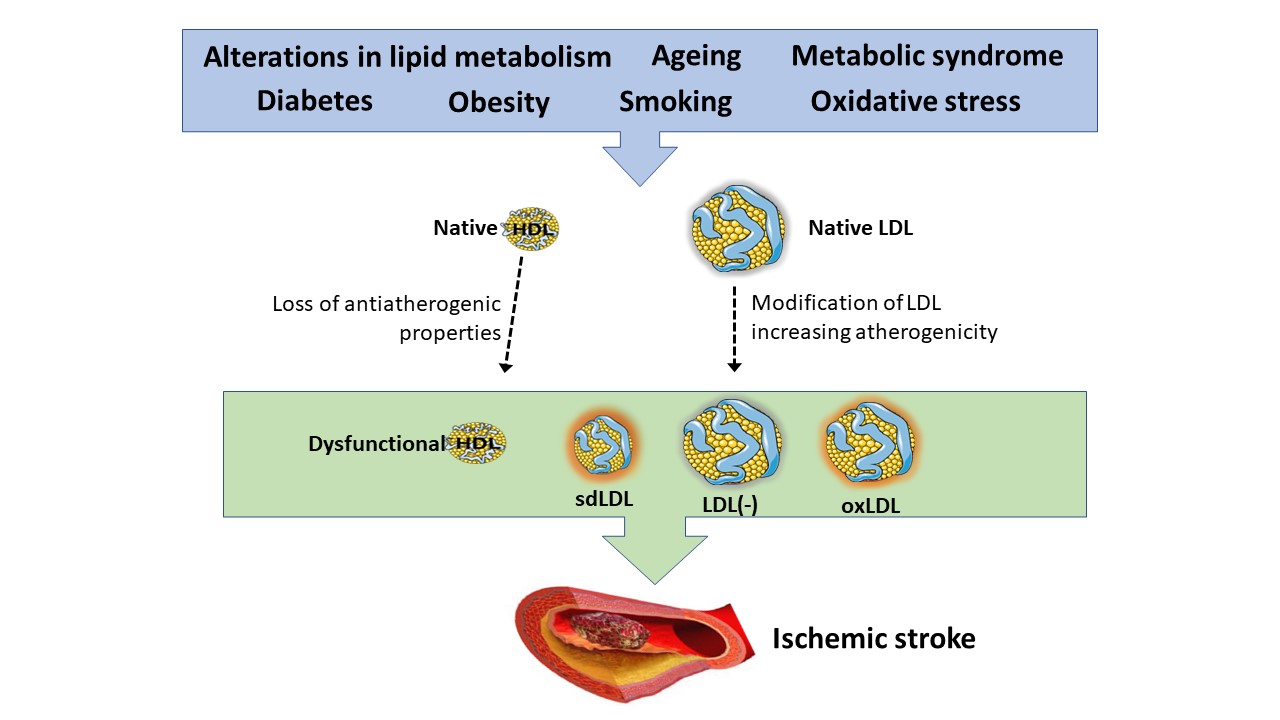

Supplement: Supplementary file 1 [file Image1.jpeg]
